# Optimizing sweet potato production: insights into the interplay of plant sanitation, virus influence, and cooking techniques for enhanced crop quality and food security

**DOI:** 10.3389/fpls.2024.1357611

**Published:** 2024-03-18

**Authors:** Anna Villalba, Eva Martínez-Ispizua, Miguel Morard, Ana Crespo-Sempere, María R. Albiach-Marti, Angeles Calatayud, Consuelo Penella

**Affiliations:** ^1^ ValGenetics S.L., Parc Científic Universitat de València, CUE-3, Paterna, Valencia, Spain; ^2^ Departamento de Horticultura, Centro de Citricultura y Producción Vegetal, Instituto Valenciano de Investigaciones Agrarias, Moncada, Valencia, Spain

**Keywords:** sweet potato *Ipomea batatas* (L.) Lam., plant sanitation, crop yield, storage root quality, cooking techniques, sustainable agriculture, viruses

## Abstract

This study investigates the impact of sweet potato plant sanitation on the yield and external and internal quality root storage exploring the nutritional content affected by various cooking methods (raw, boiled, and oven-cooked). The presence of viruses, and concretely of the sweet potato leaf curl virus (SPLCV), in sweet potato propagation material is shown to significantly reduce yield and modify storage root quality. Notably, the research reveals a substantial improvement in crop yield and external quality, reinforcing the efficacy of plant sanitation methods, specifically apical meristem culture, in preserving the overall productivity of sweet potato crops. Furthermore, the investigation identifies a noteworthy decrease in starch content, suggesting a dynamic interaction between plant sanitation and starch metabolism in response to viral diseases. The study also delves into the alteration of mineral absorption patterns, shedding light on how plant sanitation influences the uptake of essential minerals in sweet potato storage roots. While the health status of the plants only slightly affected magnesium (Mg) and manganese (Mn) accumulation, indicating a potential resilience of mineral balance under virus-infected conditions. Moreover, the research identifies significant modifications in antioxidant levels, emphasizing the role of plant sanitation in enhancing the nutritional quality of sweet potatoes. Heat-treated storage roots, subjected to various cooking methods such as boiling and oven-cooking, exhibit notable differences in internal quality parameters. These differences include increased concentrations of total soluble solids (SS) and heightened levels of antioxidant compounds, particularly phenolic and flavonoid compounds. The observed increase in antioxidant capacity underscores the potential health-promoting benefits associated with plant sanitation practices. Overall, the study underscores the critical importance of plant sanitation in enhancing sweet potato production sustainability, contributing to food security, and supporting local agricultural economies. The results emphasize the need for further research to optimize plant sanitation methods and promote their widespread adoption globally, providing valuable insights into the complex relationships in food quality.

## 1 Introduction

Sweet potato or batata (*Ipomea batatas* L.) is the third most economically important herbaceous perennial root crop worldwide (Marques et al., 2022); it is only surpassed by potato (*Solanum tuberosum* L.) and cassava (*Manihot esculenta* Crantz). Sweet potato originated from the tropical regions in South and Central America, and it was exported by the Europeans to the rest of the world. This crop is considered the seventh-ranked food raw material in the world because it is an excellent nutritional and caloric source (Monteiro et al., 2022).

Sweet potato plays important economic and nutritional roles in America, Africa, and Asia. Based on the latest statistical data, the leading annual producer is China, producing more than 50% of the world’s output (FAOSTAT, 2021; He et al., 2023). In Europe, this crop fulfills several basic roles in the global food system and sweet potato crop production is on the rise. The leading producer of this crop, amounting to 79,218.5 tons, is Spain (MAPA, 2023), followed by Portugal and Italy (FAOSTAT, 2021). In addition, lately, sweet potato has been gaining research attention. On one hand, this crop may be a potential alternative given the climate change scenario because of its moderate drought tolerance, requiring little labor, and the use of inorganic fertilizers for storage root development (Tedesco et al., 2023). Moreover, sweet potato crops have wide adaptability to marginal land, from tropical to temperate zones. On the other hand, sweet potato storage roots bear a high energy value; they give a great amount of energy output per unit area (Behera et al., 2022). Thus, they contain a variety of nutritional compounds with health-promoting properties that are suitable for the human diet and animal feed (Sanchez et al., 2020), positioning itself as a key crop to ensure food security (Truong et al., 2018).

Sweet potato storage roots contain many macronutrients including protein, lipids, dietary fiber, and carbohydrates, and micronutrients such as minerals, carotenoids, anthocyanins, phenolic acid, and vitamins (Ayeleso et al., 2016; Tanaka et al., 2017; Kourouma, 2019). Sweet potato has been classified according to their flesh color, such as purple, orange, or white (Miguel et al., 2007). The different flesh-colored varieties could determine their nutritional and phytochemical composition and change food sensory analysis. The storage roots are either used as a raw vegetable or used in cooking such as in curries, chips, breads, and cakes, as well as in biscuits, juices, ketchup, and other value-added products (Abidin et al., 2015).

The processing and cooking of sweet potatoes, mainly based on heat treatment, can lead to changes in their physical characteristics and chemical composition (Franková et al., 2022). In addition, these quality traits can also be influenced by both abiotic and biotic factors (Saito et al., 2008; Weber et al., 2015).

In relation to sweet potato phenology traits, as a result of the self-incompatibilities or the absence of production of flower, the only procedure capable of obtain ingstorage roots is by vegetative or clonal plant propagation in non-tropical climes (Monteros-Altamirano et al., 2021; Behera et al., 2022). The vegetative propagation over generations produces the accumulation of viral diseases in the crop, leading to reduced storage root yields in 80%–90%, even in the cases of asymptomatic viral diseases inducing the absence of foliar visible symptoms (Mukasa et al., 2003; Tairo et al., 2004; Adikini et al., 2016). The most important and harmful viral disease in sweet potatoes is known as sweet potato virus disease (SPVD), caused by the co-infection by the *Sweet potato chlorotic stunt virus* (SPCSV; genus *Crinivirus*, family *Closteroviridae*) and *Sweet potato feathery mottle virus* (SPFMV; genus *Potyvirus*, family *Potyviridae*) (Ogero et al., 2023). The presence of viruses significantly impacts the initial formation of storage roots, thereby directly influencing visual fruit quality characteristics. Consequently, storage roots infected with viruses may face rejection in the fresh market on a regular basis. (Clark and Hoy, 2006). For this reason, plant sanitation of infected sweet potato varieties is an essential action to preserve sweet potato crop yield. Viral particles that may be present in the plant vascular system are able to reach the meristematic region by cell-to-cell movement in a slow process (Carrington et al., 1996). Thus, when the plant is affected by pathogens, the only aerial part that is not infected is the apical meristem (Mashilo et al., 2013). Mostly due to its high metabolic activities, it is usually accompanied by elevated endogenous auxin content in shoot apices and the lack of a vascular system (Ferguson and Beveridge, 2009). Based on this finding, apical meristem culture plays an important role in eliminating the viral particles from the selected host plants. This apical meristem tissue should be cultivated in an adequate sterile growing medium under aseptic conditions to induce cell differentiation and finally obtain virus-free complete plants with sanitary assurance (Penella et al., 2020; EPPO, 2021).

Vegetable landraces in Valencia Community (eastern Mediterranean coast of Spain) such as Blanco sweet potato are demanded by consumers due to their high-quality properties, such as having white flesh and great sweetness, and their use in cooking (Miguel et al., 2007). However, its productivity is decreasing due to cycles of viral infection. This situation makes it mandatory to obtain healthy Blanco sweet potato plants through meristem-tip culture in order to preserve biodiversity. Disease-free plants are more resistant to environmental stresses and pests, resulting in higher yields and better external quality storage roots (Penella et al., 2020, 2022), leading to more sustainable and environmental-friendly crop production. Thus, the potential benefits of plant sanitation have been mentioned in some studies that demonstrated a significant increase in yield compared to the farmer’s traditional non-tested material (Clark et al., 2002; Gibson et al., 2004; Clark et al., 2010; Ling et al., 2010). However, in these studies, the effect of plant sanitation in the external and internal sweet potato storage root quality is absent. In addition, the influence of plant sanitation on the nutritional content and active biomolecules of sweet potato storage roots when they are subjected to various cooking methods is not explored. This last approach is crucial for understanding the advantages of plant sanitation in sweet potato production, especially in traditional varieties like Blanco, as it can lead to improved yield and promote local crop cultivation.

With the aim of obtaining knowledge about this issue, this study focused on determining the plant sanitation state and its effect on cooking methods in terms of production and nutritional compounds on a traditional Valencian sweet potato variety (Blanco type). To reach this objective, we have evaluated the following: (i) the differential behavior in terms of the nutritional profile and yield of Blanco sweet potato plants compared with virus-free sweet potato plants and non-sanitized plants, and (ii) whether different cooking/heat treatments commonly used in the preparation of sweet potato storage roots (raw, boiled, and oven-cooked) affect nutritional quality in either sanitized or non-sanitized plants.

## 2 Materials and methods

### 2.1 Plant material

The plant materials for this study consisted of a sweet potato [*Ipomea batatas* L (lam)] traditional variety called Blanco from the Valencia Community, Spain. The Blanco variety was provided by the Valencian Institute of Agrarian Research (IVIA, Moncada, Spain) plant germplasm bank, including two types: non-sanitized (farmer’s traditional non-viral tested material) and sanitized sweet potato plants, obtained by meristem-tip culture in previous experiments (Penella et al., 2020, 2022).

Before planting and just before the sweet potato harvest, the plant material from both sanitized and non-sanitized mother plants was evaluated for the presence of Begomovirus & Potyvirus, and Sweet potato chlorotic stunt virus (SPCSV). Total RNA and DNA was extracted from 0.25 g of fresh leaf samples using the Maxwell^®^ RSC Plant RNA Kit (Promega) and Maxwell^®^ RSC PureFood GMO Authentication Kit, respectively, according to the manufacturer’s protocol and resuspended in 50 μL of sterilized water. Both groups of RNA and DNA samples were analyzed by RT-PCR or PCR using specific primers and conditions previously reported (Marie-Jeanne et al., 2000; Li et al., 2004; Kwak et al., 2014).

### 2.2 Experimental design

Experiments were conducted from June to October 2021 in the IVIA experimental installation in Moncada (Valencia, Spain; 39°35022.3” N, 0°23044.0” W, 37 cm above sea level). Field trail was randomly designed with the sanitized as well as the non-sanitized type, in three blocks per type, including 10 plants per block. In total, 60 plants were cultivated (30 plants for each treatment). Plants were initially irrigated on-demand, closely monitoring soil moisture to maintain optimal growth conditions during the early stages of the grow cycle. Subsequently, irrigation frequency was reduced, with only two additional irrigations strategically applied throughout the remaining cycle and one rainfall peak in early October.

Sweet potatoes were harvested on 25 October 2021, and the collected usable samples correspond to the different production areas (three replicates). Sweet potatoes were weighed, and their size was measured, classifying them into two different groups: fresh market (up to 25 cm in length) and industry (more than 25 cm).

### 2.3 Visual quality determination

In order to visually determine the condition of sweet potato fruits suitable for fresh market when harvested, some of the visual quality parameters described by Huaman (1991), with slight modifications, were used as a reference. In all the cases, a single person performed the visual characterization to unify the criteria. Fruit shape and surface defects (shape, skin type, presence of veins, horizontal throttling, and/or longitudinal slits) were considered in 30 storage roots for each treatment. The purpose of this characterization was to describe the condition of the fruits from the consumer’s point of view (**Table 1**). This visual characterization was performed 1 week after harvest and before the curing process was completed. This process allows for root skin to harden and wound healing caused by handling (FAO, 2022). Sweet potatoes were placed in a greenhouse (natural light conditions with a maximum PAR of 1,000 µmol m^−2^ s^−1^, a mean temperature of 21°C, and a mean humidity of 60%) so that they could expel the latex. Sample collection consisted of nine different sweet potato fruits per treatment, for both sanitized and non-sanitized types.

**Table 1:** The rating scale for scoring the visual quality of harvested sweet potato (based on data from Huaman, 1991, with slight modifications).

Trait	Score	Description
Shape	1	Rounded or elliptic
	3	Elongated elliptic
	5	Oval, obovate, or oblong
	7	Elongated oblong or long and irregular
Vein presence	1	None
	3	Infrequent and thin
	5	Marked veins
	7	Veins very apparent
Horizontal throttling	1	None
	3	Poorly marked
	5	Marked
	7	Very marked
Longitudinal slits	1	None
	3	Poorly marked
	5	Marked
	7	Very marked

### 2.4 Internal quality assessment

#### 2.4.1 Sample preparation

**Figure 1:**
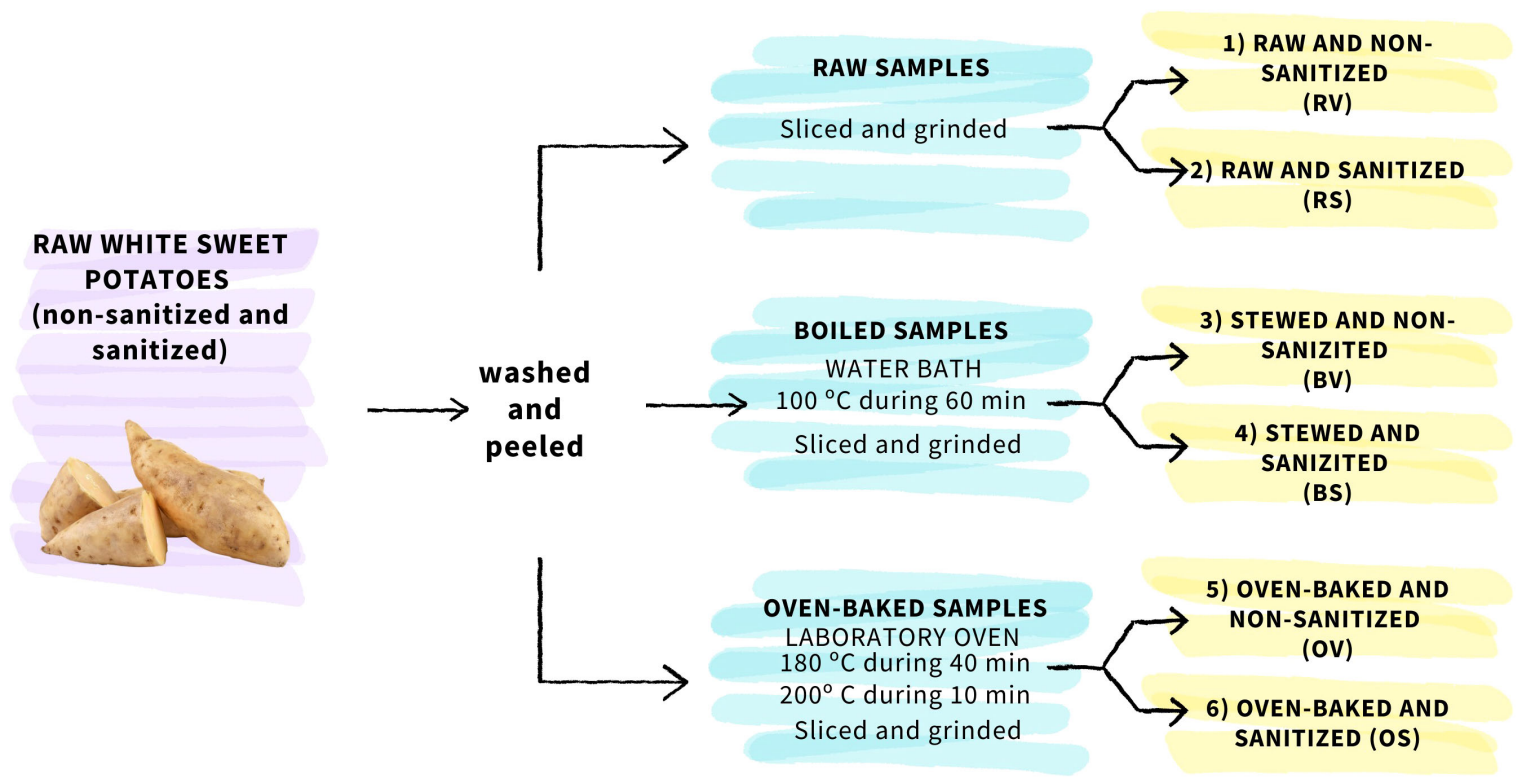
Flowchart of sweet potato sample processing.

The nutritional and nutraceutical composition in commercial sweet potatoes was determined in sanitized Blanco sweet potatoes in comparison to non-sanitized ones under various common cooking methods, including raw, boiled, and oven-baked preparations. Based on this, six different storage root groups were formed: (1) raw and non-sanitized (RV), (2) raw and sanitized (RS), (3) boiled and non-sanitized (BV), (4) boiled and sanitized (BS), (5) oven-baked and non-sanitized (OV), and (6) oven-baked and sanitized (OS) samples. Seven sweet potatoes suitable for fresh market per group were randomly selected, washed, and peeled. To investigate the effects of cooking methods, raw samples were directly stored under room conditions (20°C, 60% HR) in the absence of light, while the remaining samples were processed accordingly to the conditions indicated in **Figure 1**. Samples designated for baking were placed in a pre-heated laboratory oven (JP Selecta ™, Cincinnati, Ohio, USA), wrapped in aluminum foil, and subjected to 40 min at 180°C followed by 10 min at 200°C, to ensure uniform cooking. Samples designated for stewing were placed in a pre-heated water bath (Raypa, Terrasa, Barcelona, Spain), also covered with aluminum foil, and cooked for 60 min at 100°C to ensure optimal softness and thorough cooking.

For each cooking treatment, two slices were obtained from the central part of each storage root. Specifically, the slice selection process was conducted meticulously, ensuring that each slice was approximately 1 cm thick and using a measuring tape to guarantee uniformity. One slice was set aside for drying, while the other was cut into pieces and ground. The resulting extracts were divided into aliquots of 2 g and frozen in liquid nitrogen (−80°C) for subsequent nutritional determinations. The dried samples, reserved for mineral analysis and dry weight quantification, were processed by drying in a laboratory oven at 65°C for 72 h and subsequently ground using a mixer mill (MM400, Retsch, Hann, Germany).

#### 2.4.2 Dry weight

In order to stablish the percentage DW in storage roots, the fresh weight (FW) of storage roots was recorded. One slice from the central part of each storage root (*n* = 4) was dried at 65°C for 72 h in a laboratory oven. The DW percentage was calculated as (DW/FW) × 100.

#### 2.4.3 Mineral composition

Samples were dried in a laboratory oven at 65°C for 72 h and homogenized before being burnt in a muffle furnace for 12 h at 550°C. Macronutrients and micronutrients were extracted with 5 mL of 2% (v/v) nitric acid in an ultrasonic bath for 30 min at 40°C. Afterwards, 10 mL of 2% nitric acid was added to the solution. Specifically, the mineral concentration of eight of the main minerals related to sweet potato’s nutritional quality (K, Ca, P, Mg, Na, S, Fe, and Mn) was analyzed. Mineral content was measured by inductively coupled plasma optical emission spectrometry (ICP-OES) on an ICAP 6500 DUO/IRIS Intrepid II XLD system (Thermo Scientific, Waltham, MA, United States) (Meza et al., 2020). ICP-OES analysis was carried out at the Ionomics Platform of Centro de Edafología y Biología Aplicada del Segura (CEBAS)-Consejo Superior de Investigaciones Científicas (CSIC) (Murcia, Spain). The patterns are from Carlos Erba^®^, and the reagents used were from Panreac^®^, PA-ISO grade. The results for the macro- and micronutrients were expressed as mg g^−1^ DW and µg g^−1^ DW, respectively.

#### 2.4.4 Total soluble sugar and starch contents

Soluble sugar and starch contents were spectrophotometrically determined according to Calatayud et al. (2008), with modifications; 0.3 g of FW of the sample was mixed with 15 mL of 80% (v/v) ethanol, previously heated. The mix was incubated in a water bath for 10 min at 85°C and then vortexed. Samples were centrifuged at 10,000 rpm for 10 min at 22°C. The resulting supernatant was reserved in a flask. This same process was repeated two more times adding hot ethanol back to the mixing tube. The resulting supernatant was used for soluble sugar quantification, while the pellet was used to determine starch content.

The ethanol present in the reserved supernatant was then evaporated by means of the rotary evaporator (R-210, Buchi) at 50°C. The sugar concentrate was diluted in 100 mL of distilled water and filtered to be reserved in a volumetric flask for 24 h at 4°C. Next, 0.5 mL of this solution was reserved for further analysis.

For starch determination, 20 mL of 35% perchloric acid was added to the pellet and the opened tubes were stored in an extraction chamber for 24 h at room temperature. Afterwards, 0.05 mL of this solution was reserved for further analysis.

The last step involved running simultaneous tests for both starch and sugar content. In this experiment, 0.5 mL of the soluble sugar solution or 0.05 mL of the perchloric acid solution was mixed with 2 mL of distilled water in glass tubes and placed on ice. Once cooled, 5 mL of 4 µM anthrone solution, diluted in 96% (v/v) sulfuric acid, was added to each tube. Samples were incubated in a water bath for 7.5 min at 85°C and then placed on ice for 30 min. The absorption of the solutions was measured at 630 nm in a spectrophotometer (Lambda 25 UV/VIS, Perkin Elmer, Waltham, USA). Total soluble sugar and starch concentrations were compared with a standard curve using a diluted (1:25) stock solution of 55.6 µM glucose and 70 µM sodium benzoate. Results were expressed as g glucose equivalent 100 g^−1^ FW.

#### 2.4.5 Nutraceutical compounds and antioxidant capacity

##### 2.4.5.1 Total phenolic analysis

The total phenolic concentration was analyzed as described by Dewanto et al. (2002) with modifications. Sample extract (1 g of FW) was mixed with 4 mL of 80% (v/v) methanol, vortexed, and incubated in an ultrasonic bath (Ultrasonic cleaner, Fungilab) at medium intensity for 30 min. Samples were centrifuged at 10,000 rpm for 15 min at 4°C. Total phenolic content was determined following the Folin-Ciocalteu colorimetric method where 10 µL of the supernatant was mixed with 115 µL of distilled water, 125 µL of Folin-Ciocalteu reagent, and 1.25 mL of NaHCO_3_ (7%). This solution was vortexed and incubated at room temperature for 90 min in darkness. The absorption of the solution was measured at 760 nm in a spectrophotometer (Lambda 25 UV/VIS, Perkin Elmer, Waltham, USA). A blank solution without extract was used for calibration. Total phenolic concentration was compared to a gallic acid standard curve (120–600 mg L^-1^), and total phenolic content was expressed as mg gallic acid equivalent (GA) g^−1^ FW.

##### 2.4.5.2 Total flavonoid content

Flavonoid concentration was determined using the method reported by Du et al. (2009), with adjustments. One gram FW of sample extract was homogenized in 4.0 mL of 80% (v/v) methanol, incubated in an ultrasonic bath (Ultrasonic cleaner, Fungilab) at medium intensity for 30 min, and then vortexed. Samples were centrifuged at 10,000 rpm for 15 min at 4°C. Subsequently, 0.3 mL of the supernatant was mixed with 3.4 mL of ethanol 30%, 0.15 mL of NaNO_2_ 0.5 M, and 0.15 mL of AlCl_3_ 6H_2_O 0.3 M and then vortexed. Samples were incubated for 5 min at room temperature. Later, 1 mL of NaOH 0.1 M was added to the mixture. Subsequently, the absorption of the final solution was measured at 506 nm in a spectrophotometer (Lambda 25 UV/VIS, Perkin Elmer, Waltham, USA). Total flavonoid content was compared to a routine (PhytoLab GmbH & Co. KG^®^) standard curve (4.7–300 mg L^-1^). Flavonoid content was expressed as mg routine equivalent g^−1^ FW.

##### 2.4.5.3 Ascorbic acid concentration

Total ascorbic acid (AsA) concentration was spectrophotometrically determined according to Kampfenkel et al. (1995); 0.3 g FW of the sample was mixed with 1.5 mL with 6% (w/v) TCA (trichloroacetic acid). Samples were centrifugated at 10,000 rpm for 3 min. The supernatant (0.05 mL) was mixed with 0.05 mL of 10 mM DTT and 0.1 mL of 0.2 M phosphate buffer (pH 7.4). Then, they were incubated for 15 min at 42°C in a water bath. Afterward, 0.05 mL of 0.5% (w/v) NEM (*N*-ethylmaleimide) was added to the mix and incubated for 1 min at room temperature. Subsequently, 0.25 mL of 10% (w/v) TCA, 0.2 mL of H_3_PO_4_, 0.2 mL of 4% (w/v) 2,2´-dipyridyl, and 0.1 mL of 3% (w/v) FeCl_3_ were included to the previous solution. The final samples were incubated in a water bath for 40 min at 42°C. The absorption of the solution was measured at 525 nm in a spectrophotometer (Lambda 25 UV/VIS, Perkin Elmer, Waltham, USA). The blank solution without extract was used for calibration. Ascorbic acid was expressed as mg g^-1^ FW.

##### 2.4.5.4 Carotenoid concentrations

Total carotenoid contents were determined spectrophotometrically as reported by Porra et al. (1989), with slight adjustments; 0.3 g FW of the sample was mixed with 2.5 mL of 80% (v/v) acetone and centrifuged at 2,000 rpm for 10 min. The supernatant was used for analysis. The absorption of the solution was measured at 663.6 nm, 646.6 nm, and 470 nm in a spectrophotometer (Lambda 25 UV/VIS, Perkin Elmer, Waltham, USA). Acetone 80% (v/v) was used as the blank solution. Pigment concentrations were calculated using the following equations. (Chlorophyll a with Equation 1, chlorophyll b with Equation 2 and carotenoids with Equation 3) Results were expressed as µg g^-1^ FW:


(1) $$Chl\;a\;(\mu g\cdot mL^{-1})=12.25\times Abs663.6-2.55\times Abs646.6$$

(2) $$Chl\;b\;(\mu g\cdot mL^{-1})=20.31\times Abs646.6-4.91\times Abs663.6$$

(3) $$Carotenoids\;(\mu g\cdot mL^{-1})=[(1,000\times Abs470-1.82\times Chl\;a)-(85.02\times Chl\;b)]/198$$

##### 2.4.5.5 Antioxidant capacity measurements

Antioxidant capacity was measured using the method reported by Brand-Williams et al. (1995), with slight modifications. Sample extract (1 g) was homogenized in 4.0 mL of 80% (v/v) methanol, incubated in an ultrasonic bath (Ultrasonic cleaner, Fungilab, Barcelona, Spain) at medium intensity for 30 min, and vortexed afterward. Samples were centrifuged at 10,000 rpm for 15 min at 4°C. The supernatant (30 μL) was mixed with 990 μL of a solution composed of 0.065 M of 2,2-diphenyl-1-picrylhydrazyl (DPHH) solved in 80% (v/v) methanol. The decrease in absorbance at 515 nm compared with a blank solution [containing 80% (v/v) methanol without extract] was measured after a reaction time of 30 min at room temperature and darkness using a spectrophotometer (Lambda 25 UV/VIS, Perkin Elmer, Waltham, USA). The results were expressed as the percentage reduction of the initial DPPH absorption in the extracts.

#### 2.4.6 Statistical analysis

The normality of the distribution was verified for all studied variables using Q-Q plots, which exhibited normal behavior across all cases. Consequently, a two-way ANOVA analysis was performed with the results obtained from the evaluated parameters with Statgraphics Centurion XVIII (Statistical Graphics Corporation 2014, Englewood Cliffs, NJ, USA) using the sanitization status and the heat treatment methods as variables of the analysis. The results were expressed as the means and were compared using Fisher’s least significance difference (LSD) test at *p* ≤ 0.05.

Principal component analysis (PCA) was performed for standardized values using pairwise Euclidean distances among the means of accessions to determine similarities between sample types. The extracted and statistically significant eigenvalues and the relative and cumulative proportions of total variance explained by the first three components were calculated. Two-dimensional (2D) scatter plot (first vs. second principal components) was executed based on a distance matrix for the principal components to visualize the relation explaining the traits. Information extracted from the feature plot was incorporated into the scatter plot to highlight the traits that contributed the most to the variability among treatments. PCA parameters were estimated using the Grepel, Stat, FactoMine R, and Factoextra R packages (STHDA).

By considering quality traits, correlation analyses were completed among the sanitation status and heat treatments. The individual samples of each variable were subjected to linear regression and correlation coefficients (*r*) were obtained.

## 3 Results

### 3.1 Plant material

While non-sanitized plant material was found to be positive only for Begomovirus (sweet potato leaf curl virus, SPLCV), no virus was detected in the sanitized plant material across both measurement time points (beginning and at the conclusion of the experiment).

### 3.2 Storage root yield assessment

Sanitization process positively affects the total production of sweet potato plants, as higher values in both total and commercial production were obtained in sanitized plants (production was tripled and doubled, respectively) (**Table 2**).

**Table 2:** Blanco storage root yield assessment of harvested sweet potato.

Status	Yield evaluation		Mean ± variance (max. 10)			
Not sanitized	Total production	kg/m^2^	1.708	**±**	0.265	c
	Fresh market production	kg/m^2^	1.312	**±**	0.219	c
Sanitized	Total production	kg/m^2^	5.240	**±**	0.540	a
	Fresh market production	kg/m^2^	2.781	**±**	0.640	b

A total of 30 plants per sample type (sanitized or not) distributed in three blocks per type were subjected to the analysis. Values are the mean ± SE (n = 30 plants). The mean is subjected to a one-way ANOVA. Different lowercase letters indicate significant differences.

### 3.3 Visual quality

Quality traits related to the visual description of the harvested sweet potatoes is shown in **Table 3**, where lower values indicate a reduced occurrence of disorders, consequently enhancing consumer acceptability. Visual quality was sanitization dependent. Storage root collected from healthy plants showed an acceptable commercial shape and almost no presence of veins, horizontal throttling, or longitudinal slits. Meanwhile, non-sanitized samples showed an unmarketable storage root shape, in terms of consumer acceptance, and a higher frequency of horizontal throttling and longitudinal slits.

**Table 3:** The visual quality of harvested sweet potatoes.

Status	Descriptor punctuation	Mean ± Variance (max. 10)			
Not sanitized					
	Shape	4.67	**±**	0.34	a
	Vein presence	1.37	**±**	0.15	a
	Horizontal throttling	2.69	**±**	0.29	a
	Longitudinal slits	3.00	**±**	0.30	a
Sanitized					
	Shape	3.76	**±**	0.28	b
	Vein presence	1.55	**±**	0.13	a
	Horizontal throttling	1.78	**±**	0.24	b
	Longitudinal slits	1.90	**±**	0.24	b

A total of 30 storage roots per sample type (sanitized or not) were subjected to the analysis. The scores are rated on a 1–7 scale. Total value corresponds to the mean of all data obtained for the descriptors. Values are the mean ± SE (n = 30). The mean is subjected to a one-way ANOVA. The different lowercase letter indicates differences between status at p< 0.05 by the LSD test.

### 3.4 Internal quality

#### 3.4.1 Dry weight of storage roots

The DW of storage roots was solely influenced by the cooking method (data not shown). In its raw state, the storage roots showed a dry matter content of 30%. Baked samples displayed 25% of dry matter content, whereas boiled samples, in contrast, showed a decrease in dry matter percentage (18% dry matter) compared to the raw samples.

#### 3.3.2 Mineral composition

The mineral concentration of eight of the main minerals related to sweet potato’s nutritional quality (K, Ca, P, Mg, Na, S, Fe, and Mn) is displayed in **Table 4**.

**Table 4:** Mineral content of the harvested sweet potato samples.

Sample type	K (mg g^-1^ FW)				Ca (mg g^-1^ FW)				P (mg g^-1^ FW)				Mg (mg g^-1^ FW)			
V	16.900	±	1.337	–	1.268	±	0.334	–	1.719	±	0.163	–	0.571	±	0.053	b
S	17.343	±	1.237	–	1.258	±	0.398	–	1.768	±	0.174	–	0.660	±	0.069	a
R	17.276	±	1.133	–	1.194	±	0.181	–	1.782	±	0.113	–	0.629	±	0.071	–
B	16.536	±	1.223	–	1.468	±	0.280	–	1.699	±	0.110	–	0.620	±	0.065	–
O	17.551	±	1.405	–	1.128	±	0.271	–	1.750	±	0.250	–	0.597	±	0.045	–
RV	16.849	±	1.329	–	1.186	±	0.173	–	1.813	±	0.057	–	0.625	±	0.056	–
RS	17.704	±	0.860	–	1.200	±	0.163	–	1.750	±	0.155	–	0.718	±	0.053	–
BV	16.970	±	1.174	–	1.360	±	0.129	–	1.721	±	0.116	–	0.575	±	0.041	–
BS	16.102	±	1.269	–	1.570	±	0.189	–	1.677	±	0.117	–	0.666	±	0.052	–
OV	16.880	±	1.843	–	1.256	±	0.271	–	1.622	±	0.237	–	0.541	±	0.028	–
OS	18.222	±	0.070	–	1.001	±	0.168	–	1.879	±	0.215	–	0.596	±	0.044	–

Sample type	Na (mg g^-1^ FW)				S (mg g^-1^ FW)				Fe (mg µg^-1^ FW)				Mn (mg µg^-1^ FW)			
V	0.558	±	0.058	–	0.543	±	0.046	–	13.777	±	2.001	–	12.467	±	2.946	b
S	0.557	±	0.055	–	0.566	±	0.030	–	13.057	±	2.016	–	16.073	±	1.465	a
R	0.561	±	0.002	–	0.534	±	0.026	–	14.392	±	1.319	–	15.318	±	2.040	–
B	0.548	±	0.059	–	0.574	±	0.022	–	13.126	±	1.479	–	14.613	±	2.933	–
O	0.609	±	0.043	–	0.561	±	0.047	–	12.437	±	2.201	–	12.754	±	2.416	–
RV	0.566	±	0.043	–	0.523	±	0.033	–	14.655	±	1.214	–	15.143	±	2.664	–
RS	0.519	±	0.063	–	0.545	±	0.033	–	14.128	±	1.642	–	15.552	±	1.303	–
BV	0.566	±	0.076	–	0.560	±	0.021	–	13.806	±	1.507	–	12.329	±	2.188	–
BS	0.529	±	0.076	–	0.591	±	0.012	–	12.446	±	1.265	–	16.897	±	1.209	–
OV	0.635	±	0.024	–	0.545	±	0.059	–	12.564	±	2.373	–	10.687	±	2.738	–
OS	0.585	±	0.039	–	0.578	±	0.029	–	12.309	±	2.372	–	13.467	±	2.050	–

Values are the mean ± SE of replicates per sample type. The mean is subjected to a two-way ANOVA. Different lowercase letters indicate significant differences between (a) Not sanitized (V) vs. sanitization (S) (n = 9), (b) Cooking type [Raw (R) vs. Bath (B) vs. Oven (O)] (n = 6), (c) Interaction (Raw-not sanitized (RV), raw-sanitized (RS), boiled-not sanitized (BV), boiled-sanitized (BS), oven-cooked-not sanitized (OS), oven-cooked-sanitized (OS)) (n = 3), at p ≤ 0.05 by the LSD test. K, potassium; Ca, calcium; P, phosphorus; Mg, magnesium; Na, sodium; S, sulfur; Fe, iron; Mn, manganese.

In the case of Mg and Mn, significant results were related to the sample type [not sanitized (V) vs. sanitized (S)], with a Mg and Mn accumulation that is 13.8% and 16% higher, respectively, in the storage roots obtained from sanitized plants. No significant differences were identified among the various cooking methods. Furthermore, the interaction between sanitation and cooking method treatments was not significant in any case.

#### 3.3.3 Total soluble sugar and starch contents

##### 3.3.3.1 Total soluble sugar concentration

**Figure 2:**
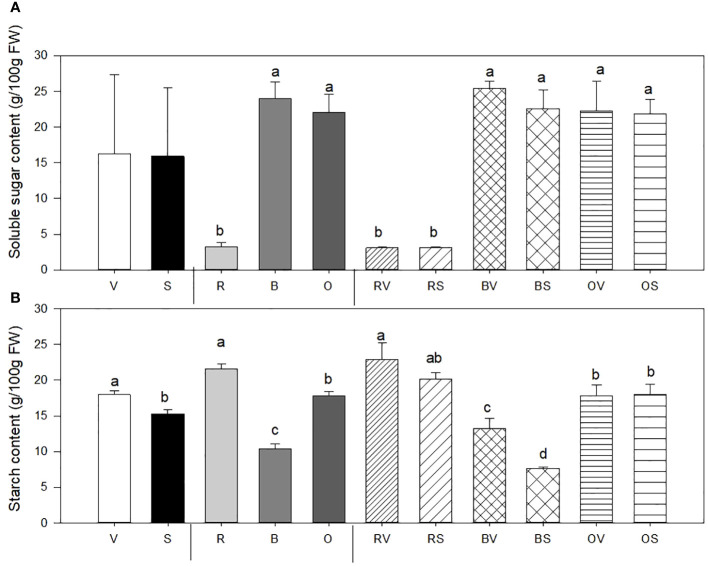
Soluble sugar content (SS) **(A)** and starch content (St) **(B)** mean values ± SE of replicates per sample type. Different lowercase letters indicate significant differences between (a) Not sanitized (V) vs. sanitization (S) (*n* = 9), (b) Cooking type [Raw (R) vs. Bath (B) vs. Oven-cooked (O)] (*n* = 6), (c) Interaction (Raw-not sanitized (RV), raw-sanitized (RS), boiled-not sanitized (BV), boiled-sanitized (BS), oven-cooked-not sanitized (OS), oven-cooked-sanitized (OS)) (*n* = 3), at *p* ≤ 0.05 by the LSD test.

Soluble sugar (SS) content in sweet potatoes is notably affected by the cooking treatment (**Figure 2A**). Boiled and oven-cooked samples show a higher SS content when compared to raw sweet potatoes (7.1 times higher SS content). By contrast, sanitization does not affect SS content in the sweet potato storage roots.

##### 3.3.3.2 Starch concentration

Starch content (St) in sweet potatoes is affected by sanitary status, cooking treatment, and interaction between sanitary condition and heat-cooking treatment (**Figure 2B**). Storage roots collected from sanitized plants exhibit a substantial 9% reduction in St content independently of heat treatment. Regarding the cooking method, boiled sweet potatoes displayed a decrease in starch content compared to their raw and oven-baked counterparts (56.45% and 23.27%, respectively). The interaction between sanitary status and cooking/heat treatment showed statistical differences only on boiled samples. Moreover, the lowest values were detected in sanitized and boiled samples (43.75% lower content compared with those non-sanitized and boiled), and the highest value was held by raw and non-sanitized storage roots (RV), followed by those raw and sanitized (RS) without significant differences between them.

### 3.4 Nutraceutical compounds and antioxidant capacity

#### 3.4.1 Total phenolic concentration

**Figure 3:**
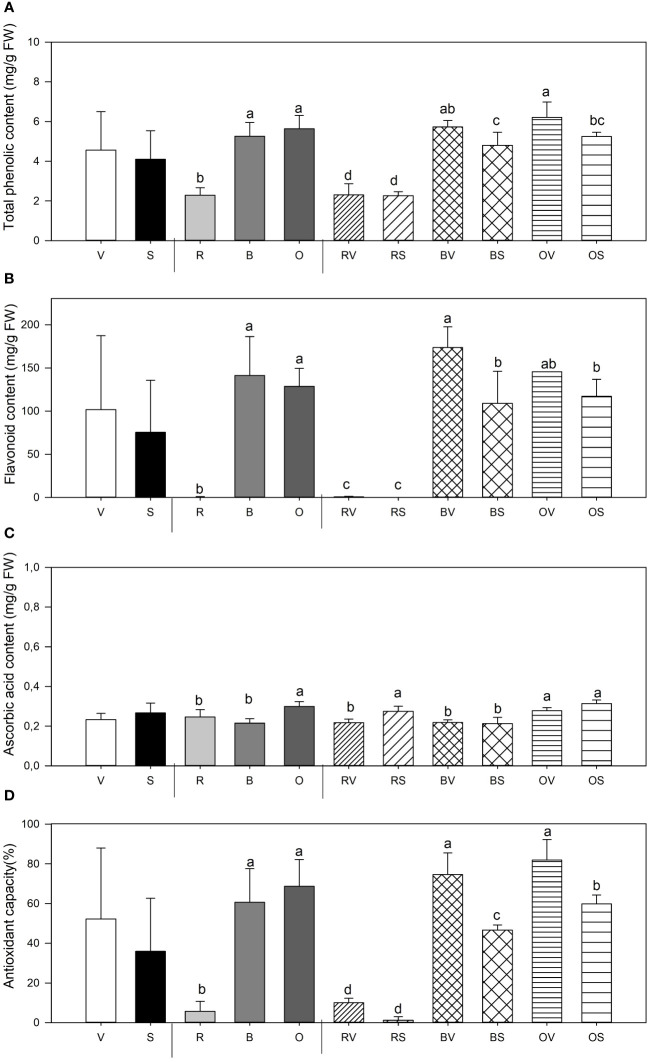
Total phenolic (Phe) content **(A)**, flavonoid content (Flav) **(B)**, ascorbic acid content (vitamin C) **(C)** and antioxidant capacity (%) **(D)** mean values ± SE of replicates per sample type. Different lowercase letters indicate significant differences between (a) Not sanitized (V) vs. sanitization (S) (*n* = 9), (b) Cooking type [Raw (R) vs. Bath (B)vs. Oven (O)] (*n* = 6), (C) Interaction (Raw-not sanitized (RV), raw-sanitized (RS), boiled-not sanitized (BV), boiled-sanitized (BS), oven-cooked-not sanitized (OS), oven-cooked-sanitized (OS)) (*n* = 3), at *p ≤* 0.05 by the LSD test.

Considerable total phenolic (Phe) content differences were found depending on the cooking method used and its interaction with sanitation state (**Figure 3A**). Heat treatment, both boiling (B) and oven-cooked (O), significantly increased the concentration of Phe content, duplicating the concentration in sweet potatoes [2.36 and 2.40 times higher content, respectively, than the mean of the raw samples (R)]. Concerning raw samples, sanitation treatment does not affect Phe content.

#### 3.4.2 Total flavonoid content

Sweet potatoes’ flavonoid (Flav) content is significantly affected by thermal treatment and the interaction among both studied variables, plant sanitation and cooking method (**Figure 3B**). While uncooked samples had little or no content of Flav, boiled and oven sweet potatoes presented a higher concentration. A greater accumulation of flavonoids in boiled virulent samples is shown in comparison with boiled storage roots collected from healthy plants. No differences were found in raw and baked samples depending on their health status.

#### 3.4.3 Ascorbic acid concentration

Significant differences among sanitary status, thermal treatment, and its interaction were found concerning AsA content in samples analyzed (**Figure 3C**). Storage roots collected from sanitized plants showed the highest AsA content. Moreover, cooking method is relevant to establish vitamin C concentration, as oven-cooked sweet potatoes presented the highest AsA values when compared to raw and boiled ones [21.54% (O vs. R) and 39.1% (O vs. B) higher, respectively]. Meristem-tip culture favored the accumulation of vitamin C only in the raw samples, with no such differences observed in the thermally treated samples among them.

#### 3.4.4 Antioxidant capacity determination

Antioxidant capacity, as determined by the DPPH assay, was dependent on thermal treatment and its interaction with sample sanitation status (**Figure 3D**). Raw samples showed the lowest antioxidant capacity when compared to boiled and oven-cooked samples. In relation to the interaction of both parameters, cooking treatments and being non-sanitized increased DPPH content in both sample types. Conversely, such effect cannot be noted on raw samples.

#### 3.4.5 Carotenoid concentrations

**Figure 4:**
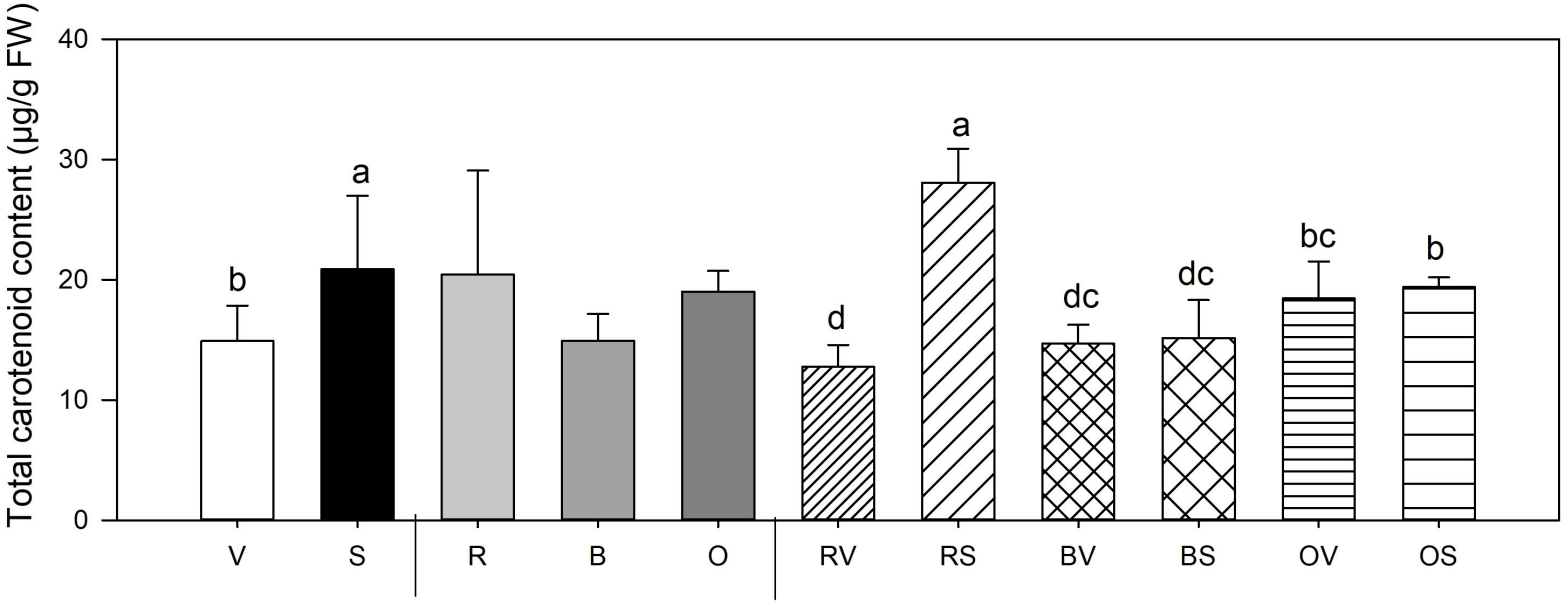
Total carotenoid mean values ± SE of replicates per sample type. Different lowercase letters indicate significant differences between a) Not sanitized (V) vs sanitization (Sanitized samples (S)) (n=9) b) Cooking type (Raw (R) vs Bath (B) vs Oven (O)) (n=6) c) Interaction (Raw-not sanitized (RV), raw-sanitized (RS), boiled-not sanitized (BV), boiled-sanitized (BS), oven-cooked-not sanitized (OS), oven-cooked-sanitized (OS)) (n=3), at p≤0.05 by the LSD test.

Concerning carotenoid concentration, significant differences between sanitation status and its interaction with heat treatments were found (**Figure 4**). Sanitized storage roots accumulate more carotenoid content in their fruits (40% higher concentration than storage roots from non-sanitized plants). When considering the interaction of both variables, health status and cooking method, this effect is exclusively observed in the raw storage roots, with the highest values associated to plant’s sanitation state with no discernible differences in the storage roots that were thermally treated.

### 3.5 Correlation between internal quality parameters

Correlation analyses were carried out to estimate the relation between the most important quality traits in the sweet potato storage roots (**Table 5**). The most representative relations were observed between several compounds, such as Flav, AsA, DPPH, SS, St and K, P, and Mg minerals.

**Table 5:** Linear correlation coefficient (*r*) and its significance among the quality traits in sweet potato samples.

	Car	Phe	Flav	AsA	DPPH	SS	St	K	Ca	P	Mg
Car		-0.3934*	-0.3745	0.4772*	-0.3992	-0.3554	0.2245	0.4854*	-0.4992*	0.1196	0.4729*
Phe			0.9337***	-0.0778	0.9544***	0.9055***	-0.3377	-0.14	0.4065	-0.3171	-0.5114*
Flav				-0.0173	0.9566***	0.9179***	-0.4537*	-0.2524	0.2596	-0.0301	-0.5175*
AsA					-0.0571	-0.0734	0.4165*	0.3996	-0.6502**	0.1317	-0.1962
DPPH						0.9047***	-0.2705	0.0742	0.3733	-0.211	-0.6546**
SS							-0.5453	-0.085	0.2841	-0.1697	-0.5903**
St								0.382	-0.5731*	0.4041	-0.1632
K									-0.5452**	0.4894*	0.0261
Ca										-0.2324	-0.0732
P											0.1023
Mg											

DW, dry weight; Car, carotenoids; Phe, phenols; Flav, flavonoids; AsA, ascorbic acid; DPPH, antioxidant capacity; SS, soluble sugars; St, starch; K, potassium; Ca, calcium; P, phosphorus; Mg, magnesium.

In bold, statiscally significant correlations have been highlighted. ***, **, and * indicate significance at *p ≤* 0.001, *p ≤* 0.01, and *p ≤* 0.05.

Among samples, the pairwise coefficients showed a positive correlation and a statistical significance for 11 out of the 55 studied pairs of traits. Important positive relations were observed between Flav, DPPH, Phe, and SS, with the highest values observed for the pairs DPPH vs. Flav and Phe (*r* = 0.9566 and *r* = 0.9544, respectively). Statistically significant negative correlations were also observed for 10 of the 55 studied pairs of traits. The closest negative relations were observed for DPPH and Mg (*r* = −0.6546), AsA vs. Ca (*r* = −0.6502), and St and Ca (*r* = −0.5452).

### 3.6 Global analysis of the internal quality parameters

**Figure 5:**
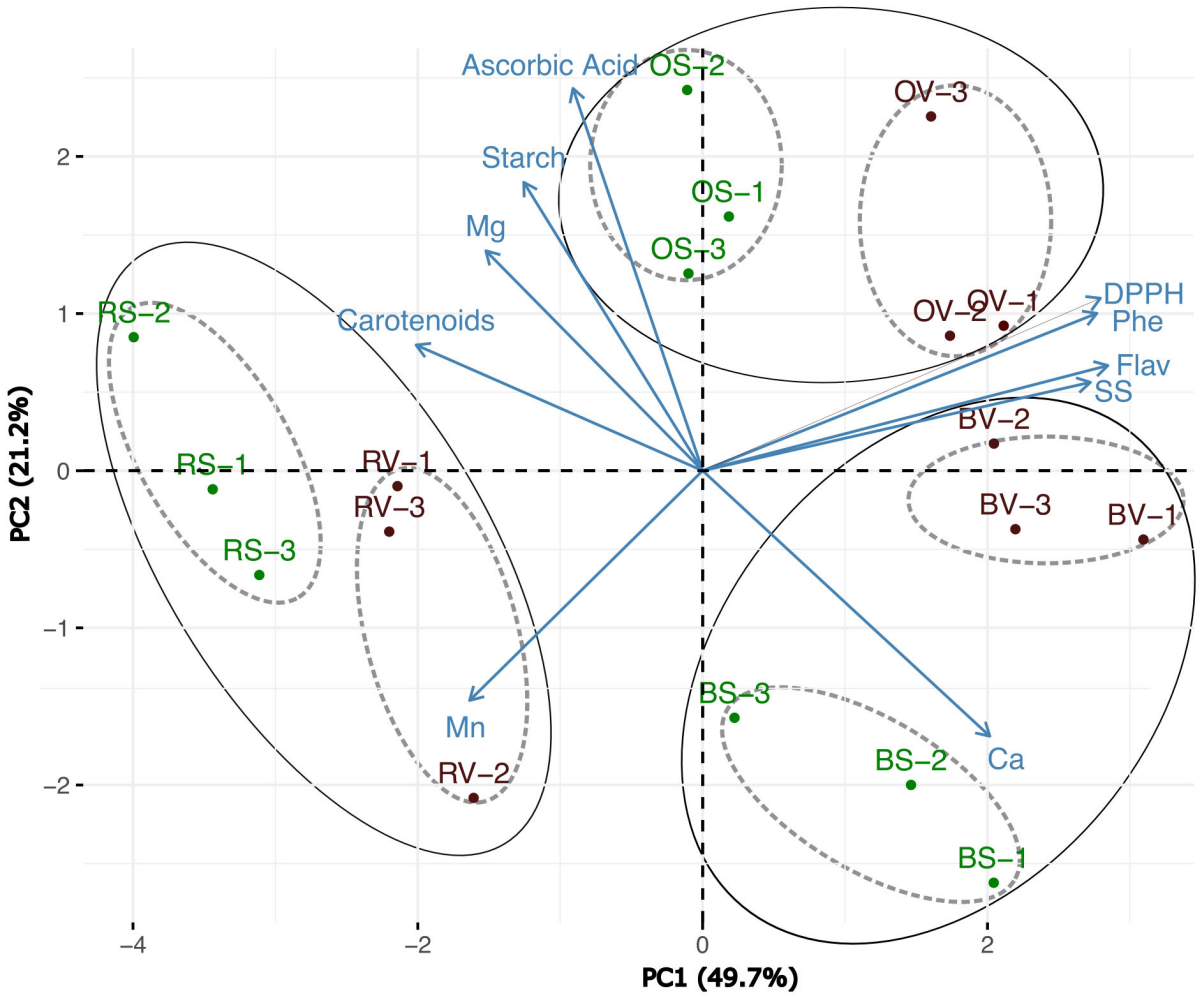
Principal component analysis (PCA) based on quality traits represented in the 2 first components of the PCA for the type of cooking and sanitation process (R, raw; B, boiled; O, oven-baked; S, sanitized V, virulent). A total of 6 storage root for sample type were subjected to the analysis.

A principal component analysis was performed on the complete dataset of nutraceutical and antioxidant parameters (**Figure 5**). The PCA clustered the samples with high confidence, accounting for 70.9% of the total variability (49.7% with PC1 and 21.2% with PC2). The clustering divides the samples into three broad groups that are distinguished by the heat-cooking method. The samples exhibit a subclustering phenomenon within these general clusters, which is driven by the sanitation status of the samples. Independent PCAs (**Supplementary Figures S1A–C**) were made to determine if the samples clustered belonging to the heat treatment could show a differential response. The PCA related to raw treatment (**Supplementary Figure S1A**) was explained by 67% of the total variability (43% the first component and 24% the second component). PCA related to the boiling process (**Supplementary Figure S1B**) was explained by 70.3% (44% for the first component and 26.3% for the second component), and differences in oven-cooked samples (**Supplementary Figure S1C**) were 78.6% of the total variability (57.8% for the first component and 20.8% for the second component). In all cases studied, the samples exhibited effective clustering, grouped in sanitized and non-sanitized ones. Depending on the cooking methods applied, various characteristics were associated with either sanitized or non-sanitized samples (**Supplementary Figure S1**). In general, traits related to the antioxidant capacity (DPPH, flavonoids, and total phenolic content); starch and calcium were predominantly associated with non-sanitized samples. Conversely, traits such as AsA, magnesium, and manganese were prominently linked to sanitized samples. In the case of raw and oven-baked samples within the sanitized group, soluble sugars were also identified as a significant trait.

## 4 Discussion

The detrimental effects of planting pathogen-infected material constitute one of the major problems that are directly impacting crop viability. This study has documented that the presence of viruses in the propagation plant material significantly diminished yield and modified storage root quality.

The experiments performed in this research have demonstrated that the plant sanitation process promotes a successful production with a remarkable threefold increase in potential yield of sanitized sweet potato plants compared with non-sanitized plants. Similarly, the commercial production of the Blanco-type landrace has effectively doubled planting sanitized plants. These findings align with previous field trials conducted with popular and heirloom cultivars (e.g., Beauregard, Georgia Jet, Hernandez, etc.) (Milgram et al., 1996; Bryan et al., 2003b; Clark and Hoy, 2006; Ling et al., 2010), indicating that yield improvements can be achieved by employing virus-free materials for planting. Other studies have reported similar effects on crops such as potato and cassava (Whitworth et al., 2007; Al-Taleb et al., 2011; Samura et al., 2017).

Furthermore, the evidence gathered from this experiment sheds light on the considerable potential of plant sanitation practices for enhancing sweet potato quality. Storage root collected from healthy plants exhibited a commercially acceptable shape with minimal presence of veins, horizontal throttling, or longitudinal slits. Similar effects were observed in Beauregard sweet potatoes infected with SPLCV (Bryan et al., 2003a; Clark and Hoy, 2006), where infection resulted in a decrease in commercial yield due to the development of shallow and longitudinal grooves. The significant improvement in visual quality observed in the Blanco-type sanitized landrace highlights the economic benefits of implementing this approach in crop cultivation systems.

In a second objective, we analyzed the interaction between sanitation status and standard cooking processes. As expected, the cooking process dramatically influences the quality of storage root. However, the specific implications of virus presence or absence in plants on storage root internal quality have largely remained unexplored until now. This study has demonstrated that some storage root internal quality parameters are influenced by both the use of sanitized and non-sanitized plants, plus the chosen cooking method. The cooking method generally showed a more pronounced impact on storage root quality, explaining 49.7% of the variability, although there were exceptions. In a PCA, clear groupings were observed based on sanitary condition after categorizing samples by cooking methods, emphasizing the importance of plant sanitation for nutritional and nutraceutical quality.

In a broader context, heat-treated storage roots exhibited a significant increase in internal quality parameters (except in starch and carotenoids). Boiling and oven-cooking significantly increased the total SS content, which aligns with previous findings showing that cooking reduces starch content through amylolytic hydrolysis, leading to an increase in reducing sugar contents (Wei et al., 2017; Nicoletto et al., 2018; Yao et al., 2023; Zhang et al., 2023). Notably, recent investigations by Moreno-Ochoa et al. (2023) in sweet potato have revealed that an increase in cooking temperature is associated with a decrease in starch content.

Phenolic and flavonoid compounds, which are major antioxidants in plant-based foods, were influenced also by cooking methods (Turkmen et al., 2005), affecting their retention and bioavailability in storage roots. In our experiments, Phe and Flav analysis indicated the increase of the antioxidant capacity of the cooked storage roots, as a result of the presence of higher amounts of all antioxidant compounds (in general terms, 10 times more in cooked samples compared to control). These findings are in close agreement with those reported by Kim et al. (2019), who observed high Phe and Flav content in heat-treated sweet potatoes compared with raw storage roots, likely due to the thermal processing damaging cell structure. This cell structure damage results in the extraction of antioxidant compounds and the partial removal of water from the tissue samples during the cooking process (Nicoletto et al., 2018), or from the skin, and it has been observed in tomato (Dewanto et al., 2002). The lower water content in cooked storage roots may further reduce dilution effects, enhancing the effectiveness of antioxidant compounds against oxidative stress.

When assessing AsA content, the oven-cooked samples exhibited the highest concentrations (34.78% increase in AsA content compared with raw and boiled samples), a trend consistent with earlier findings reported by Chukwu et al. (2012) and Inocent et al. (2011) in thermal processed sweet potatoes. These findings align with various studies, such as the work of Nicoletto et al. (2018), which highlighted a significantly reduced vitamin C content in comparison with control, whereas steaming results did not show differences from controls. Similar observations have been made in the context of potatoes, where cooking processes involving water, including washing, were found to facilitate the diffusion of vitamin C into the treatment water, as noted by Haase and Weber (2003). Remarkably, our study underscores that the oven method resulted in the most significant concentration of vitamin C, likely attributable to a concentration effect, where water dilution comes into play.

In contrast, cooking sweet potato storage roots reduced the carotenoid content (Calderón de la Barca et al., 2022; Moreno-Ochoa et al., 2023). Carotenoids are easily oxidized because of conjugated double bonds found in the compounds (Krinsky, 2001). Reduction in carotenoid content could be explained by the fact that carotenoids are heat-labile compounds and undergo oxidation and degradation upon exposure to heat, light, acids, peroxides, metals, and enzymes (K’osambo et al., 1998; Gul et al., 2015).

In summary, the cooking process significantly affects the content of antioxidants and other essential nutrients in sweet potatoes, including starch, carotenoids, and AsA. Furthermore, the presence or absence of viruses in the plant material has a clear impact not only on external quality but also on the internal quality of storage roots, with sanitized plants showing notable differences when compared with control non-sanitized storage roots using the same cooking method. Ultimately, the nutritional parameters affected by sanitation were mineral absorption, starch content, carotenoids, and AsA.

Effects of virus infection in the plant can include damage to the root system, interference with nutrient transport, changes in mineral metabolism (Mishra et al., 2020), and disruption of hormonal regulation (Llave, 2016), promoting an imbalance between the leaf (source) and the root system (Anikina et al., 2023). These changes can lead to nutrient deficiencies, affecting the growth and development of sweet potato storage roots (Clark et al., 2012; Ogero et al., 2023). In our study, health status only slightly affected the Mg and Mn accumulation in storage roots, indicating that infection did not deeply alter the ionic balance in our conditions. In fact, mineral nutrition in the soil may slow or exacerbate the effect of virus in mineral plant uptake (Barker, 2019). The soils utilized in our experiment exhibit high fertility levels, potentially mitigating the anticipate negative effects on ion imbalance in plant tissues associated with virus presence. This response was also observed in other horticultural crops such as rutabaga (Shattuck, 1987) and eggplant (Seaker et al., 1982) under virus infection.

Regarding starch, previous studies have demonstrated that in response to abiotic stresses, plants mobilize their starch reserves to release energy, sugar, and derived metabolites (Krasensky and Jonak, 2012; Thalmann and Santelia, 2017; Dong and Beckles, 2019). However, the effects of starch metabolism on biotic stress responses remain unclear (Llave, 2016). Under non-sanitation virus conditions, the Blanco-type sweet potato plants seem to respond by modulating their starch reserves. Starch content has been reported to be positively correlated with the degree of infection in potato inoculated with *Alternaria alternata* (Fladung and Gieffers, 1993). Substantial starch accumulation has also been observed in *Plasmodiophora brassicae* infected Chinese cabbage roots at 21 and 28 days after infection (Ma et al., 2022), and a similar effect was seen in *Arabidopsis thaliana* roots infected by pathogens (Brodmann et al., 2002). Similarly, a recent study conducted on an Asian variety called Jish n°6 (Hou et al., 2020) revealed a reduction in starch content in roots of plants severely infected with SPLCV. Although there were no significant differences observed in plants with moderate infection compared to the control plants, these findings collectively suggest notable implications in relation to infections caused by virulent diseases and starch metabolism.

In the case of Blanco sweet potatoes, our results reveal that starch metabolism is affected by SPLCV viral disease. These findings insinuate a dynamic interaction between starch metabolism and the presence of viruses in plant responses, thus indicating a potential link between the severity of the SPLCV and alterations in starch reserves. These observations also emphasize the possible significance of varietal effects in SPLCV infections and highlights the need for further research studies to fully comprehend the intricacies of starch metabolism in the impact of biotic stressors.

According to Seel et al. (2020), pathogens induced alteration in pigment content (carotenoids) as well as divergent behaviors of plant secondary metabolism, such as AsA content (Oladiran and Iwu, 1992), along with defense activation. This is consistent with the observations made in this study, where pathogen infection led to a reduction in carotenoid and AsA contents in storage roots from diseased plants. Some studies have indicated a positive correlation between AsA and total carotenoids in other food matrices, such as watermelon, spinach, or even fruit beverages (Bergquist et al., 2007; Butcher et al., 2012). In fact, the SPLCV disease has been attributed to potentially acting as a blocker in carotenoid biosynthesis (Hou et al., 2020). The presence of SPLCV disease in Blanco sweet potato plants not only impacts the pigment content and the AsA levels but also affects the potential synergistic relationship between these antioxidants such as demonstrated by correlation between both parameters.

To recapitulate the analyses of all the samples collectively, intriguing associations emerged between Ca, Mg, and K mineral levels and bioactive compounds. For example, it was observed that Ca exhibited negative correlations with certain bioactive compounds, such as carotenoids (*r* = −0.4992, *p* ≤ 0.05) and AsA (*r* = −0.6502, *p* ≤ 0.01), Mg with phenolic content (*r* = −0.5114, *p* ≤ 0.05), and flavonoid content (*r* = −0.5175, *p* ≤ 0.05), and a positive effect among K and carotenoids content was found (*r* = 0.4854, *p* ≤ 0.05). These findings are significant as they provide insights into the potential interactions between mineral content and bioactive compounds in the tested samples. The negative correlations between certain minerals and specific bioactive compounds may have implications for food and nutrition research. Further investigations are warranted to understand the underlying mechanisms driving these associations. Additionally, the significance of the observed correlations should be considered in the context of other factors that may influence mineral and bioactive compound interactions, such as sample origin, processing methods, and overall dietary patterns. Overall, these results contribute to the growing body of knowledge regarding the complex relationships between mineral levels and bioactive compounds in food, enhancing our understanding of the potential health impacts of dietary choice when vegetal materials come from plant sanitized crops.

## 5 Conclusions

In summary, it is evident that the planting of sanitized plants and the presence of viruses, particularly SPLCV in the sweet potato propagation material, exert a significant influence, not only on crop yield but also on both the external and internal quality of the storage roots. This influence extends to the choice of cooking method employed, as it plays a pivotal role in shaping the overall quality of the final food product. The observed effects encompass a range of crucial parameters, including alterations in starch content, modifications in mineral absorption patterns, fluctuations in AsA levels, and variations in carotenoid concentrations. Understanding and carefully considering the interplay between sanitized plants, virus’ presence, cooking techniques, and these key quality attributes is of paramount importance in ensuring the production of nutritionally superior and safe food products.

The findings from this study reinforce the importance of plant sanitation techniques as an essential component of modern agricultural practices. By effectively mitigating the impact of viruses and other pathogens, this approach offers a sustainable and efficient strategy for meeting the increasing demand for sweet potato production. The adoption of such practices can not only bolster crop yields but also contribute to promoting food security and sustaining local agricultural economies. Further research and extension efforts in this direction are warranted to optimize the implementation of plant sanitation methods and to foster their widespread adoption in sweet potato cultivation worldwide.

## Data availability statement

The original contributions presented in the study are included in the article/supplementary materials, further inquiries can be directed to the corresponding author/s.

## Author contributions

AV: Data curation, Formal analysis, Methodology, Visualization, Writing – original draft, Writing – review & editing. EM-I: Data curation, Formal analysis, Methodology, Visualization, Writing – original draft, Writing – review & editing. MM: Data curation, Formal analysis, Validation, Visualization, Writing – original draft, Writing – review & editing. AC-S: Methodology, Validation, Writing – original draft. MA-M: Funding acquisition, Project administration, Resources, Supervision, Writing – review & editing. AC: Conceptualization, Funding acquisition, Investigation, Methodology, Project administration, Resources, Supervision, Validation, Writing – original draft, Writing – review & editing. CP: Conceptualization, Data curation, Formal analysis, Investigation, Methodology, Supervision, Validation, Visualization, Writing – original draft, Writing – review & editing.

## References

[B1] Abidin P. E. Dery E. Amagloh F. Asare K. Amoaful E. Carey E. (2015). Training of Trainers’ Module for Orange-Fleshed Sweetpotato (OFSP). Utilization and processing. International Potato Center (CIP); Nutrition Department of the Ghana Health Service, Tamale (Ghana). 32pp. doi: 10.4160/9789290604624

[B2] Adikini S. Mukasa S. B. Mwanga R. O. M. Gibson R. W. (2016). Effects of sweet potato feathery mottle virus and sweet potato chlorotic stunt virus on the yield of sweetPotato in Uganda. J. Phytopathol. 164, 242–254. doi: 10.1111/JPH.12451

[B3] Al-Taleb M. M. Hassawi D. S. Abu-Romman S. M. (2011). Production of virus free potato plants using meristem culture from cultivars grown under Jordanian environment. J. Agric. Environ. Sci. 11, 467–472. Available at: https://www.semanticscholar.org/paper/Production-of-Virus-Free-Potato-Plants-Using-from-Al-Taleb-Hassawi/cca890d18210bb075ca4b85e3a230e1c9ce06536.

[B4] Anikina I. Kamarova A. Issayeva K. Issakhanova S. Mustafayeva N. Insebayeva M.. (2023). Plant protection from virus: a review of different approaches. Front. Plant Sci. 14. doi: 10.3389/FPLS.2023.1163270 PMC10291191 37377807

[B5] Ayeleso T. B. Ramachela K. Mukwevho E. (2016). A review of therapeutic potentials of sweet potato: Pharmacological activities and influence of the cultivar. Trop. J. Pharm. Res. 15, 2751–2761. doi: 10.4314/TJPR.V15I12.31

[B6] Barker A. V. (2019). Mineral nutrition and plant disease. HortScience 44, 1510a. doi: 10.21273/HORTSCI.44.5.1510A

[B7] Behera S. Chauhan V. B. S. Pati K. Bansode V. Nedunchezhiyan M. Verma A. K.. (2022). Biology and biotechnological aspect of sweet potato (Ipomoea batatas L.): a commercially important tuber crop. Planta 256 (2), 40. doi: 10.1007/s00425-022-03938-8 35834064

[B8] Bergquist S. A. M. Gertsson U. E. Nordmark L. Y. G.. (2007). Effects of shade nettings, sow- ing time and storage on baby spinach flavonoids. J. Sci. Food Agric. 87, 2464–2471.

[B9] Brand-Williams W. Cuvelier M. E. Berset C. (1995). Use of a free radical method to evaluate antioxidant activity. LWT. - Food Sci. Technol. 28, 25–30. doi: 10.1016/S0023-6438(95)80008-5

[B10] Brodmann D. Schuller A. Ludwig-Müller J. Aeschbacher R. A. Wiemken A. Boller T.. (2002). Induction of trehalase in arabidopsis plants infected with the trehalose-producing pathogen plasmodiophora brassicae. Mol Plant Microbe Interact. 15, 693–700. doi: 10.1094/MPMI.2002.15.7.693 12118885

[B11] Bryan A. D. Pesic-VanEsbroeck Z. Schultheis J. R. Pecota K. V. Swallow W. H. Yencho G. C. (2003a). Cultivar decline in sweetpotato: I. Impact of micropropagation on yield, storage root quality, and virus incidence in “Beauregard”. J. Am. Soc Hortic. Sci. 128, 846–855. doi: 10.21273/JASHS.128.6.0846

[B12] Bryan A. D. Schultheis J. R. Pesic-VanEsbroeck Z. Yencho G. C. (2003b). Cultivar decline in sweetpotato: II. Impact of virus infection on yield and storage root quality in “Beauregard” and “Hernandez”. J. Am. Soc Hortic. Sci. 128, 856–863. doi: 10.21273/JASHS.128.6.0856

[B13] Butcher J. D. Crosby K. M. Yoo K. S. Patil B. S. Ibrahim A. M. H. Leskovar D. I.. (2012). Environmental and Genotypic Variation of Capsaicinoid and Flavonoid Concentrations in Habanero (Capsicum chinense) Peppers. HortScience horts. 47 (5), 574–579. doi: 10.21273/HORTSCI.47.5.574

[B14] Calatayud Á. Gorbe E. Roca D. Martínez P. F. (2008). Effect of two nutrient solution temperatures on nitrate uptake, nitrate reductase activity, NH4+ concentration and chlorophyll a fluorescence in rose plants. Environ. Exp. Bot. 64, 65–74. doi: 10.1016/j.envexpbot.2008.02.003

[B15] Calderón de la Barca A. M. Porras-Loaiza M. A. P. Pineda-Díaz E. A. González-Ríos H. Heredia-Sandoval N. G. Islas-Rubio A. R. (2022). Wheat-free and nutritious bread and ‘Coricos’ Made with mesoamerican ancestral corn, amaranth, sweet potato and chia. Plant Foods. Hum. Nutr. 77, 591–598. doi: 10.1007/s11130-022-01005-x 35987934

[B16] Carrington J. C. Kasschau K. D. Mahajan S. K. Schaad M. C. (1996). Cell-to-cell and long-distance transport of viruses in plants. Plant Cell 8, 1669. doi: 10.1105/TPC.8.10.1669 12239357 PMC161306

[B17] Chukwu O. Nwadike N. Nwachukwu N. G. (2012) Academic research international Effects of cooking and frying on antioxidants present in sweet potatoes (ipomoea batatas). Available online at: www.savap.org.pkwww.journals.savap.org.pk (Accessed November 20, 2023).

[B18] Clark C. A. Davis J. A. Abad J. A. Cuellar W. J. Fuentes S. Kreuze J. F.. (2012). Sweetpotato viruses: 15 years of progress on understanding and managing complex diseases. Plant Dis. 96, 168–185. doi: 10.1094/PDIS-07-11-0550 30731810

[B19] Clark C. A. Hoy M. W. (2006). Effects of common viruses on yield and quality of beauregard sweetpotato in Louisiana. Plant Dis. 90, 83–88. doi: 10.1094/PD-90-0083 30786480

[B20] Clark C. A. Smith T. P. Ferrin D. M. Villordon A. Q. (2010). Performance of sweetpotato foundation seed after incorporation into commercial operations in Louisiana. Horttechnology 20, 977–982. doi: 10.21273/HORTSCI.20.6.977

[B21] Clark C. A. Valverde R. A. Fuentes S. Salazar L. F. Moyer J. W. (2002). Research for improved management of sweetpotato pests and diseases: Cultivar decline. Acta Hortic. 583, 103–112. doi: 10.17660/ACTAHORTIC.2002.583.11

[B22] Dewanto V. Xianzhong W. Adom K. K. Liu R. H. (2002). Thermal processing enhances the nutritional value of tomatoes by increasing total antioxidant activity. J. Agric. Food Chem. 50, 3010–3014. doi: 10.1021/JF0115589 11982434

[B23] Dong S. Beckles D. M. (2019). Dynamic changes in the starch-sugar interconversion within plant source and sink tissues promote a better abiotic stress response. J. Plant Physiol. 234–235, 80–93. doi: 10.1016/J.JPLPH.2019.01.007 30685652

[B24] Du G. Li M. Ma F. Liang D. (2009). Antioxidant capacity and the relationship with polyphenol and Vitamin C in Actinidia fruits. Food Chem. 113, 557–562. doi: 10.1016/J.FOODCHEM.2008.08.025

[B25] EPPO. (2021). Production of healthy plants for planting., (2021). European and mediterranean plant Protection Organization. Available online at: https://www.eppo.int/RESOURCES/eppo_standards/pm4_certification (Accessed 2023-07-3).

[B26] FAO. (2022). Word Food and Agriculture. Statistical Yearbook 2022 (Rome). Available online at: https://www.fao.org/3/cc2211en/cc2211en.pdf (Accessed 2023-10-2).

[B27] FAOSTAT. (2021). Production: Crops and livestock products (Rome: FAO). Available at: https://www.fao.org/faostat/en/#data/QCL.

[B28] Ferguson B. J. Beveridge C. A. (2009). Roles for auxin, cytokinin, and strigolactone in regulating shoot branching. Plant Physiol. 149, 1929. doi: 10.1104/PP.109.135475 19218361 PMC2663762

[B29] Fladung M. Gieffers W. (1993). Resistance reactions of leaves and tubers of rolC transgenic tetraploid potato to bacterial and fungal pathogens. Correlation with sugar, starch and chlorophyll content. Physiol. Mol. Plant Pathol. 42, 123–132. doi: 10.1006/PMPP.1993.1010

[B30] Franková H. Musilová J. Árvay J. Šnirc M. Jančo I. Lidiková J.. (2022). Changes in antioxidant properties and phenolics in sweet potatoes (Ipomoea batatas L.) due to heat treatments. Molecules 27, 1884. doi: 10.3390/MOLECULES27061884 35335244 PMC8950918

[B31] Gibson R. W. Aritua V. Byamukama E. Mpembe I. Kayongo J. (2004). Control strategies for sweet potato virus disease in Africa. Virus Res. 100, 115–122. doi: 10.1016/j.virusres.2003.12.023 15036842

[B32] Gul K. Tak A. Singh A. K. Singh P. Yousuf B. Abas A.. (2015). Chemistry, encapsulation, and health benefits of β-carotene-A review. Cogent. Food Agric. 1, 1018696. doi: 10.1080/23311932.2015.1018696

[B33] Haase N. U. Weber L. (2003). Ascorbic acid losses during processing of French fries and potato chips. J. Food Eng. 56, 207–209. doi: 10.1016/S0260-8774(02)00252-2

[B34] He H. J. Wang Y. Wang Y. Ou X. Liu H. Zhang M. (2023). Towards achieving online prediction of starch in postharvest sweet potato [Ipomoea batatas (L.) Lam] by NIR combined with linear algorithm. J. Food Compos. Anal. 118, 105220. doi: 10.1016/J.JFCA.2023.105220

[B35] Hou F. Xie B. Qin Z. Li A. Dong S. Zhang H.. (2020). Sweetpotato leaf curl virus decreased storage root yield and quality of edible sweetpotato in China. Agron. J. 112, 3948–3962. doi: 10.1002/AGJ2.20025

[B36] Huaman Z. (1991). Electrophoretic characterization of potato germplasm at CIP. In: International Potato Center (CIP). Molecular Methods for Potato Improvement. Report of the Planning Conference on 'Application of Molecular Techniques to Potato Germplasm Enhancement', Lima, Perú, 5-9 March 1990. Lima, Perú: International Potato Center., p. 129–133.

[B37] Inocent G. Adelaide D. M. Giséle E. L. Solange M. O. R. Richard E. A. Elie F. (2011). Impact of three cooking methods (steaming, roasting on charcoal and frying) on the β-carotene and vitamin C contents of plantain and sweet potato. Am. J. Food Technol. 6, 994–1001. doi: 10.3923/AJFT.2011.994.1001

[B38] Kampfenkel K. Van Montagu M. Inzé D. (1995). Extraction and determination of ascorbate and dehydroascorbate from plant tissue. Anal. Biochem. 225, 165–167. doi: 10.1006/ABIO.1995.1127 7778771

[B39] Kim M. Y. Lee B. W. Lee H. U. Lee Y. Y. Kim M. H. Lee J. Y.. (2019). Phenolic compounds and antioxidant activity in sweet potato after heat treatment. J. Sci. Food Agric. 99, 6833–6840. doi: 10.1002/JSFA.9968 31385299

[B40] K’osambo L. M. Carey E. E. Misra A. K. Wilkes J. Hagenimana V. (1998). Influence of age, farming site, and boiling on pro-vitamin A content in sweet potato (Ipomoea batatas(L.) lam.) storage roots. J. Food Compos. Anal. 11, 305–321. doi: 10.1006/JFCA.1998.0591

[B41] Kourouma V. (2019). Comparative study on chemical composition, polyphenols, flavonoids, carotenoids and antioxidant activities of various cultivars of sweet potato. Int. J. Food Sci. Technol 104, 134–141. doi: 10.1016/j.lwt.2019.01.011

[B42] Krasensky J. Jonak C. (2012). Drought, salt, and temperature stress-induced metabolic rearrangements and regulatory networks. J. Exp. Bot. 63, 1593–1608. doi: 10.1093/JXB/ERR460 22291134 PMC4359903

[B43] Krinsky N. I. (2001). Carotenoids as antioxidants. Nutrition 17, 815–817. doi: 10.1016/S0899-9007(01)00651-7 11684386

[B44] Kwak H. R. Kim M. K. Shin J. C. Lee Y. J. Seo J. K. Lee H. U.. (2014). The current incidence of viral disease in korean sweet potatoes and development of multiplex RT-PCR assays for simultaneous detection of eight sweet potato viruses. Plant Pathol. J. 30, 416. doi: 10.5423/PPJ.OA.04.2014.0029 25506306 PMC4262294

[B45] Li R. Salih S. Hurtt S. (2004). Detection of geminiviruses in sweetpotato by polymerase chain reaction. Plant Dis. 88, 1347–1351. doi: 10.1094/PDIS.2004.88.12.1347 30795196

[B46] Ling K. S. Jackson D. M. Harrison H. Simmons A. M. Pesic-VanEsbroeck Z. (2010). Field evaluation of yield effects on the U.S.A. heirloom sweetpotato cultivars infected by Sweet potato leaf curl virus. Crop Prot. 29, 757–765. doi: 10.1016/J.CROPRO.2010.02.017

[B47] Llave C. (2016). Dynamic cross-talk between host primary metabolism and viruses during infections in plants. Curr. Opin. Virol. 19, 50–55. doi: 10.1016/J.COVIRO.2016.06.013 27442236

[B48] MAPA. (2023). Superficies y producciones anuales de cultivo. Available at: https://www.mapa.gob.es/es/estadistica/temas/estadisticas-agrarias/agricultura/superficies-producciones-anuales-cultivos/ (Accessed on 22 November 2024).

[B49] Ma Y. Choi S. R. Wang Y. Chhapekar S. S. Zhang X. Wang Y.. (2022). Starch content changes and metabolism-related gene regulation of Chinese cabbage synergistically induced by Plasmodiophora brassicae infection. Hortic. Res. 9, uhab071. doi: 10.1093/HR/UHAB071 35043157 PMC9015896

[B50] Marie-Jeanne V. Ioos R. Peyre J. Alliot B. Signoret P. (2000). Differentiation of Poaceae potyviruses by reverse transcription-polymerase chain reaction and restriction analysis. J. Phytopathol. 148, 141–151. doi: 10.1046/J.1439-0434.2000.00473.X

[B51] Marques T. S. Moreira R. F. A. Ayres E. M. M. (2022). Characterization of the essential oils from leaves of different sweet potato cultivars grown in Brazil. South Afr. J. Bot. 144, 18–22. doi: 10.1016/J.SAJB.2021.09.005

[B52] Mashilo J. Van Niekerk R. Shanahan P. (2013). Combined thermotherapy and meristem tip culture for efficient elimination of Feathery mottle virus in sweet potato (Ipomoea batatas). Acta Hortic. 1007, 719–726. doi: 10.17660/ACTAHORTIC.2013.1007.83

[B53] Meza S. L. R. Egea I. Massaretto I. L. Morales B. Purgatto E. Egea-Fernández J. M.. (2020). Traditional tomato varieties improve fruit quality without affecting fruit yield under moderate salt stress. Front. Plant Sci. 11. doi: 10.3389/fpls.2020.587754 PMC7701295 33304365

[B54] Miguel A. de la Torre F. Baixauli C. Maroto J. V. Jordá M. C. López M. M.. (2007). Boniato. Cultivos hortícolas al aire libre (Valencia, Spain: Ministerio de Agricultura, Pesca y Alimentación y Fundación Rural Caja).

[B55] Milgram M. Cohen J. Loebenstein G. (1996). Effects of sweet potato feathery mottle virus and sweet potato sunken vein virus on sweet potato yields and rates of reinfection of virus-free planting material in Israel. Phytoparasitica 24, 189–193. doi: 10.1007/BF02981417

[B56] Mishra J. Srivastava R. Trivedi P. K. Verma P. C. (2020). Effect of virus infection on the secondary metabolite production and phytohormone biosynthesis in plants. 3 Biotech. 10, 547. doi: 10.1007/S13205-020-02541-6 PMC7683645 33269181

[B57] Monteiro R. L. Oliveira de Moraes J. Gomide A. I. Mattar Carciofi B. A. Laurindo J. B. (2022). Temperature control for high-quality oil-free sweet potato CHIPS produced by microwave rotary drying under vacuum. LWT 157, 113047. doi: 10.1016/J.LWT.2021.113047

[B58] Monteros-Altamirano A. Paredes D. Buitrón-Bustamante J. Tapia C. Peña G. (2021). Genetic diversity of sweet potatoes [Ipomoea batatas (L) Lam.] in Ecuador. Genet. Resour. Crop Evol. 68, 307–320. doi: 10.1007/S10722-020-00987-4

[B59] Moreno-Ochoa M. F. Calderón de la Barca A. M. Cárdenas-López J. L. Robles-Sánchez R. M. Rouzaud-Sández O. (2023). Technological properties of orange sweet potato flour intended for functional food products as affected by conventional drying and milling methods. ACS Food Sci. Technol. 3, 283–291. doi: 10.1021/ACSFOODSCITECH.2C00308

[B60] Mukasa S. B. Rubaihayo P. R. Valkonen J. P. T. (2003). Incidence of viruses and viruslike diseases of sweetpotato in Uganda. Plant Dis. 87, 329–335. doi: 10.1094/PDIS.2003.87.4.329 30831824

[B61] Nicoletto C. Vianello F. Sambo P. (2018). Effect of different home-cooking methods on textural and nutritional properties of sweet potato genotypes grown in temperate climate conditions. J. Sci. Food Agric. 98, 574–581. doi: 10.1002/JSFA.8499 28653506

[B62] Ogero K. Okuku H. S. Wanjala B. McEwan M. Almekinders C. Kreuze J.. (2023). Degeneration of cleaned-up, virus-tested sweetpotato seed vines in Tanzania. Crop Prot. 169, 106261. doi: 10.1016/J.CROPRO.2023.106261

[B63] Oladiran A. O. Iwu L. N. (1992). Changes in ascorbic acid and carbohydrate contents in tomato fruits infected with pathogens. Plant Foods. Hum. Nutr. 42, 373–382. doi: 10.1007/BF02194098/METRICS 1438080

[B64] Penella C. Cervera M. Marsal Peset J. I. Crespo Sempere A. Albiach Martí M. R. Calatayud Chover Á. (2020). El boniato: una hortaliza tradicional en crecimiento exponencial. Agrícola. Vergel. Frutic. Hortic. Floric. 428, 202–208.

[B65] Penella C. Marsal J. I. Villalba A. González-Martínez A. Morard M. Crespo-Sempere A.. (2022). Recuperación del cultivo de boniato. Necesidad del saneamiento de las plantas frente a virus. Available online at: https://www.bibliotecahorticultura.com/publicaciones/tecnicas-de-cultivo/sanidad-vegetal/recuperacion-del-cultivo-de-boniato/.

[B66] Porra R. J. Thompson W. A. Kriedemann P. E. (1989). Determination of accurate extinction coefficients and simultaneous equations for assaying chlorophylls a and b extracted with four different solvents: verification of the concentration of chlorophyll standards by atomic absorption spectroscopy. BBA. - Bioenerg. 975, 384–394. doi: 10.1016/S0005-2728(89)80347-0

[B67] Saito T. Matsukura C. Ban Y. Shoji K. Sugiyama M. Fukuda N.. (2008). Salinity stress affects assimilate metabolism at the gene-expression level during fruit development and improves fruit quality in tomato (Solanum lycopersicum L.). J. Japanese. Soc Hortic. Sci. 77, 61–68. doi: 10.2503/JJSHS1.77.61

[B68] Samura A. E. Lakoh K. A. Nabay O. Fomba S. N. Koroma J. P. C. (2017). Effect of cassava mosaic disease (CMD) on yield and profitability of cassava and gari production enterprises in Sierra Leone. J. Agric. Sci. 9, 205. doi: 10.5539/JAS.V9N2P205

[B69] Sanchez P. D. C. Hashim N. Shamsudin R. Mohd Nor M. Z. (2020). Applications of imaging and spectroscopy techniques for non-destructive quality evaluation of potatoes and sweet potatoes: A review. Trends Food Sci. Technol. 96, 208–221. doi: 10.1016/J.TIFS.2019.12.027

[B70] Seaker E. M. Bergman E. L. Romaine C. P. (1982). Effects of magnesium on tobacco mosaic virus-infected eggplants1. J. Am. Soc Hortic. Sci. 107, 162–166. doi: 10.21273/JASHS.107.1.162

[B71] Seel W. Baust D. Sons D. Albers M. Etzbach L. Fuss J.. (2020). Carotenoids are used as regulators for membrane fluidity by Staphylococcus xylosus. Sci. Rep. 10, 330. doi: 10.1038/S41598-019-57006-5 31941915 PMC6962212

[B72] Shattuck V. I. (1987). Effect of turnip mosaic virus infection on the mineral composition of rutabaga. Commun. Soil Sci. Plant Anal. 18, 1269–1279. doi: 10.1080/00103628709367898

[B73] Tairo F. Kullaya A. Valkonen J. P. T. (2004). Incidence of viruses infecting sweetpotato in Tanzania. Plant Dis. 88, 916–920. doi: 10.1094/PDIS.2004.88.9.916 30812241

[B74] Tanaka M. Ishiguro K. Oki T. Okuno S. (2017). Functional components in sweetpotato and their genetic improvement. Breed. Sci. 67, 52–61. doi: 10.1270/JSBBS.16125 28465668 PMC5407917

[B75] Tedesco D. Moreira B. R. de A. Barbosa Júnior M. R. Maeda M. da Silva R. P. (2023). Sustainable management of sweet potatoes: A review on practices, strategies, and opportunities in nutrition-sensitive agriculture, energy security, and quality of life. Agric. Syst. 210, 103693. doi: 10.1016/J.AGSY.2023.103693

[B76] Thalmann M. Santelia D. (2017). Starch as a determinant of plant fitness under abiotic stress. New Phytol. 214, 943–951. doi: 10.1111/NPH.14491 28277621

[B77] Truong V. D. Avula R. Y. Pecota K. V. Yencho G. C. (2018). Sweetpotato production, processing, and nutritional quality. Handb. Veg. Veg. Process. Second. Ed. 2–2, 811–838. doi: 10.1002/9781119098935.CH35

[B78] Turkmen N. Sari F. Velioglu Y. S. (2005). The effect of cooking methods on total phenolics and antioxidant activity of selected green vegetables. Food Chem. 93, 713–718. doi: 10.1016/J.FOODCHEM.2004.12.038

[B79] Weber N. Veberic R. Mikulic-Petkovsek M. Stampar F. Koron D. Munda A.. (2015). Metabolite accumulation in strawberry (Fragaria × ananassa Duch.) fruits and runners in response to Colletotrichum nymphaeae infection. Physiol. Mol. Plant Pathol. 92, 119–129. doi: 10.1016/J.PMPP.2015.10.003

[B80] Wei S. Lu G. Cao H. (2017). Effects of cooking methods on starch and sugar composition of sweetpotato storage roots. PloS One 12 (8), e0182604. doi: 10.1371/JOURNAL.PONE.0182604 28827808 PMC5565179

[B81] Whitworth J. L. Nolte P. McIntosh C. Davidson R. (2007). Effect of potato virus Y on yield of three potato cultivars grown under different nitrogen levels. Plant Disease 90, 73–76. doi: 10.1094/PD-90-0073 30786478

[B82] Yao Y. Zhang R. Jia R. Deng Y. Wang Z. (2023). Impact of different cooking methods on the chemical profile of orange-fleshed sweet potato (Ipomoea batatas L.). LWT 173, 114288. doi: 10.1016/J.LWT.2022.114288

[B83] Zhang R. Chen H. Chen Y. Tang C. Jiang B. Wang Z. (2023). Impact of different cooking methods on the flavor and chemical profile of yellow-fleshed table-stock sweetpotatoes (Ipomoea batatas L.). Food Chem. X. 17, 100542. doi: 10.1016/j.fochx.2022.100542 36824146 PMC9941418

