# Supplementary figures and images for: Optimizing sweet potato production: insights into the interplay of plant sanitation, virus influence, and cooking techniques for enhanced crop quality and food security
Source: Front Plant Sci. 2024 Mar 18;15:1357611. doi: 10.3389/fpls.2024.1357611 (PMC10983796; doi:10.3389/fpls.2024.1357611)

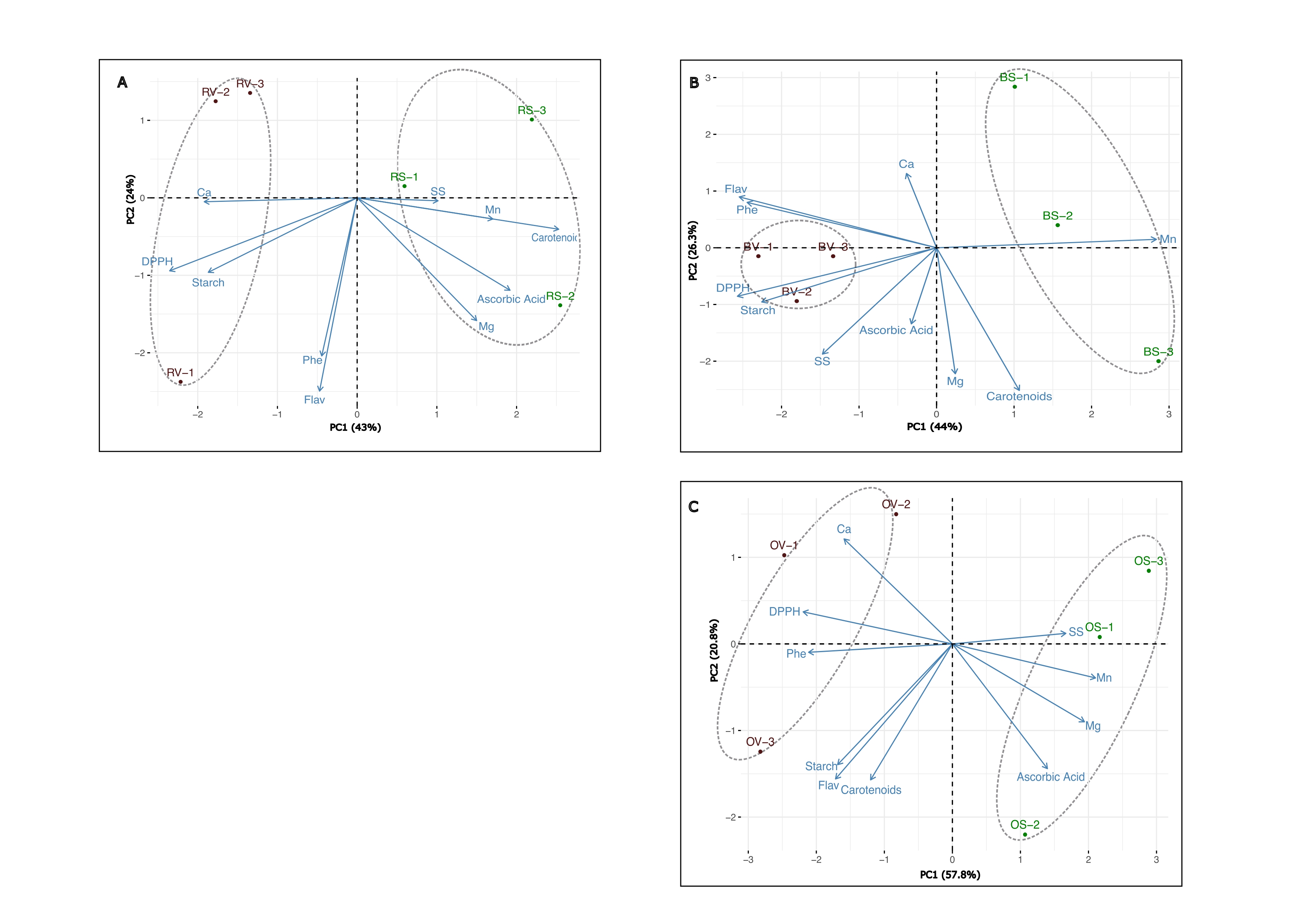

Supplement: Supplementary file 1 [file Image_1.jpg]
